# Effectiveness of capecitabine with or without docetaxel therapy for the treatment of patients with advanced urothelial carcinoma: a single-institution experience

**DOI:** 10.18632/oncotarget.11641

**Published:** 2016-08-26

**Authors:** Cong Xue, Xin An, Ye Cao, Tanhuan Chen, Wei Yang, Yingfei Deng, Hui Han, Xiaoyu Teng, Fangjian Zhou, Yanxia Shi

**Affiliations:** ^1^ Department of Medical Oncology, State Key Laboratory of Oncology in South China, Collaborative Innovation Center for Cancer Medicine, Sun Yat-Sen University Cancer Center, Guangzhou, People's Republic of China; ^2^ Department of GCP, State Key Laboratory of Oncology in South China, Collaborative Innovation Center for Cancer Medicine, Sun Yat-Sen University Cancer Center, Guangzhou, People's Republic of China; ^3^ Department of Urology, State Key Laboratory of Oncology in South China, Collaborative Innovation Center for Cancer Medicine, Sun Yat-Sen University Cancer Center, Guangzhou, People's Republic of China

**Keywords:** capecitabine, docetaxel, second-line, chemotherapy, urothelial cancer

## Abstract

**Purpose:**

The purpose of this study was to evaluate the effectiveness and toxicity of capecitabine (C) chemotherapy regimen with or without (w/o) docetaxel (D) in patients with advanced urothelial carcinoma (UC).

**Results:**

Clinical benefit rate were similar in two arms (C arm vs DC arm: 38.9% vs 45.5%, *p* = 0.411). There were two cases achieved partial response in DC arm. In C arm, the median PFS was 3.0 months (95% CI 2.5–3.5 months) and median OS was 11.3 months (95% CI 8.6–14.1 months). In DC arm, the median PFS was 2.2 months (95% CI 1.7–2.7 months) and median OS was 18 months (95% CI 6.8–29.9 months). Adverse events were mostly acceptable, including myelosuppession, hand-foot syndrome and mucositis. Anemia and leukopenia was found more in the DC arm than in the C arm.

**Materials and Methods:**

This is a one-center, observational, retrospective study. From April 2009 to March 2015, a total of 29 patients with metastatic UC were included in the study. Survivals, response rates and toxicities were collected retrospectively.

**Conclusions:**

The result showed the activity and toxicity of C w/o D. As DC treatment did not reveal better outcome, C or D single-agent might be an option in platinum-failed patients with advanced urothelial carcinoma. Further clinical trials are warranted.

## INTRODUCTION

Urothelial cancer (UC) is a common cancer in men in China [1]. The prognosis of patients with advanced UC is quite poor, with median overall survival (OS) of 10–15 months [2–4]. The standard first-line treatment for metastatic UC is cisplatin combined with gemcitabine, for which response rate is about 50%, with a median progression-free survival (PFS) of 7–8 months. Dose- dense MVAC is also a standard regimen with similar response rate and more toxicity [3]. Once the disease progress the response rate (RR) of palliative regimen is usually less than 20%, while remaining survival of the patient is quite short (usually 6–9 months) [5].

Till now there are no standard palliative regimens for patients who failed platinum-based therapy. The optional second-line agents include taxanes (docetaxel and paclitaxel) [6, 7], pemetrexed [8–10], vinflunine [11, 12] and so on. Single-agent regimen is preferred for palliative chemotherapy. Although higher RR is attained in combination therapy, the high RR seldom translate into an improvement in survivals (especially OS) [5]. The toxicity is even greater and intolerable in pretreated patients. Therefore, it is essential to find an ideal second-line therapy for metastatic UC.

Capecitabine (C), an orally bioavailable 5-fluorouracil (FU) prodrug, is widely used in solid tumors for its wide-spectrum efficacy, mild toxicity and oral convenience [13–15]. The combination of 5-FU and other chemotherapy agents had been assessed in metastatic UC. Most regimens were active and tolerable [16–20]. Capecitabine is an optional radiosensitizing agent which given during concurrent radiation as well [21]. However little is known about using capecitabine in advanced UC, including single-agent or combination therapy.

We conducted a retrospective study to investigate the clinical efficacy and toxicity of capecitabine in advanced UC. As docetaxel (D) is a commonly used second-line agent in metastatic UC [6], moreover docetaxel and capecitabine (DC) is a proved effective and tolerable regimen in metastatic breast cancer [13], the DC combination regimen is seldom reported in UC. Therefore, we also reviewed and analyzed the data of DC therapy. To our knowledge, no study has evaluated the clinical usage of capecitabine with or without (w/o) docetaxel in advanced UC. Thus, the primary objective of this retrospective observational study was to evaluate the efficacy and toxicity profile of capecitabine w/o docetaxel chemotherapy regimen, in order to provide another treatment choice of patients with advanced UC.

## RESULTS

A total of 29 patients were included in the study. All were with good performance status (ECOG 0-1). Eighteen patients were in the C arm and 11 patients were in the DC arm. Their characteristics are listed in Table 1. In the whole group, most patients were men. More male Patients in the C arm were found than those in the DC arm (*p* = 0.033). Other characteristics were balanced between two arms. 20.7% (*n* = 6) had liver metastases and 51.7% (*n* = 15) had visceral metastases (lung, liver, or brain). Prior gemcitabine and platinum exposure were recorded in 24 patients, including 21 for the first-line, and 3 for the adjuvant chemotherapy. Five patients who received DC or C as first line therapy were chemotherapy-naïve. Twenty-two patients had transitional-cell carcinomas (TCC). The other histologies included adenocarcinoma (*n* = 3), signet ring cell carcinoma (*n* = 1), and poorly differentiated carcinoma (*n* = 3).

**Table 1:** Baseline patients characteristics

Characteristic	C arm (*n* = 18)		DC arm (*n*= 11)		*p*
	No.	%	No.	%	
Age, years					
Median	53.5		59		
Range	31–82		28–70		
Sex					0.033
Male	17	94.4	7	63.6	
Female	1	5.6	4	36.4	
Histology					0.558
TCC	13	72.2	9	81.8	
Others	5	27.8	2	18.2	
ECOG					0.577
0	11	61.1	5	45.5	
1	7	38.9	6	54.5	
Time from prior therapy					0.853
≤ 3 months	10		5	45.5	
> 3 months	8		6	54.5	
Visceral metastases					0.812
Yes	9	50	6	54.5	
No	9	50	5	45.5.	
Liver metastases					0.494
Yes	3	16.7	3	27.3	
No	15	83.3	8	72.7	
Primary invasive tumor site					0.293
Bladder	10	55.6	7	63.6	
Renal and upper urinary tract	5	27.8	5*	36.4	
Urachus	3	16.7	/	/	0.976
Line of therapy					
First line	5	27.8	3	27.2	
Second line	13	72.2	8	72.8	
Prior gemcitabine and platinum exposure					0.364
Yes	14	77.8	10	90.9	
Adjuvant GP	1	5.6	2	18.2	
First line GP	13	72.2	8	72.7	
No	4	22.2	1	9.1	
Prior nephrectomy					0.229
Yes	3	16.7	4	36.4	
No	15	83.3	7	63.6	

* One patient was diagnosed with bladder and pelvic ureteral carcinoma. TCC, Transitional-cell carcinoma.

Median follow-up time was 6.53 months (range, 1.3 to 41.2 months). At the time of analysis, 27 patients had progressed, 13 patients had died. The median maintenance period of capecitabine was 59 days. The median number of docetaxel regimens administered was 2 (range, 2–4).

Statistically significant difference of clinical benefit rate (CBR) was not found between patients in C arm (7/18, 38.9%) and those in DC arm (5/11, 45.5%, *p* = 0.411). Partial response (PR) was achieved in two patients in DC arm (2/11, 18.2%). None of patients in C arm achieved PR.

When capecitabine was administrated alone, the median PFS was 3.0 months (95% CI 2.5–3.5 months) and median OS was 11.3 months (95% CI 8.6–14.1 months). When in DC combination the median PFS was 2.2 months (95% CI 1.7–2.7 months) and median OS was 18 months (95% CI 6.8–29.9 months). The differences of PFS and OS between two arms were similar and had no statistical significance (PFS, *p* = 0.810; OS, *p* = 0.771).

Subgroup analysis showed that the prognosis of patients with non-TCC was significantly poorer than those with TCC. PFS of TCC was 4.8 months (95% CI 0.9–8.7 months) while non-TCC was 1.8 months (95% CI 1.6–2.1 months, *p* = 0.003). OS of TCC was 11.3 months (95%CI 3.9–18.7 months), significantly longer than that of non-TCC, 4.1 months (95% CI 1.4–6.7 months, *p* = 0.004).

Dose reduction of capecitabine was required in five patients for the following reasons: hand-foot syndrome (HFS, two, one in C arm and one in DC arm), mucositis (two in C arm), and leukopenia (one in DC arm). One patient in the C arm required treatment discontinuation because of thrombopenia. Dose reduction of docetaxel was required in one patient for edema. Two patients required treatment discontinuation because of edema (*n* = 1) and leukopenia (*n* = 1).

The most common adverse events (AEs) are listed in Table 2. All of them were consistent with the known toxicity of capecitabine and docetaxel. The most commonly reported AE was anemia. And this event happened more in the DC arm than in the C arm (81.2% vs 22.2%, *p* = 0.002). Other reported AEs included leukopenia, mucositis, thrombocytopenia and hand-foot syndrome. Among them, leukopenia happened more in the DC arm than in the C arm as well (63.6% vs 11.1%, *p* = 0.003). The frequencies of other AEs were similar between two arms. Most of these AEs were mild. The most common grade 3 or worse AEs was leukopenia (*n* = 2) and thrombocytopenia (*n* = 1). Both happened in the DC arm. But the differences between two arms were not statistically significant.

**Table 2:** Adverse events

Adverse event	C arm (*n* = 18)				DC arm (*n* =11)			
	Grade							
	Any		3 or 4		Any		3 or 4	
	No.	%	No.	%	No.	%	No.	%
Anemia	4	22.2	–	–	9	81.2	–	–
Leukopenia	2	11.1	–	–	7	63.6	2	18.2
Mucositis	2	11.1	–	–	3	27.3	–	–
Thrombocytopenia	1	5.6	–	–	1	9.9	1	9.9
Hand-foot skin reaction	1	5.6	–	–	3	16.7	–	–
Anorexia	2	11.1	–	–	2	18.2	–	–
Edema	–	–	–	–	2	18.2	–	–
Fatigue	1	5.6	–	–	1	9.9	–	–
Diarrhea	–	–	–	–	1	9.9	–	–
ALT elevation	1	5.6	–	–	–	–	–	–

ALT: alanine aminotransferase.

We tried to evaluate the association of following factors with OS, like hemoglobin, albumin, time from prior therapy, liver metastasis, neutrophil, lymphocyte, platelet counts and performance status by Cox regression model. These factors were formerly found to be associated with OS [22–24]. However all factors were not associated with OS in univariate analysis (all *p* > 0.05). So we did not perform multivariate analysis afterwards.

## DISCUSSION

The efficacy and toxicity of patients treated with capecitabine w/o docetaxel for advanced UC was reported for the first time. The major limitations of this study are the retrospective nature and, limited sample size and immature survival data. Further randomized trials are warranted.

Results of a phase II study showed that a RR of 15% and median PFS of 1.9 months of continuously infused 5-FU in metastatic UC, indicating the prolonged 5-FU administration might be a useful regimens for these patients [16]. Capecitabine converts into 5-FU via thymidine phosphorylase (TP) in the tumor. Previous study showed that the expression of TP was generally high in UC, indicating the potential usage of capecitabine in UC [25]. In our study the PFS of 3.0 months of capecitabine single agent was similar to former studies [10]. Although there was no major response (CR or PR) in the population with single agent, the survivals were not shorter than those with combination treatment, indicating single-agent might be an eligible choice in the second-line setting.

Former *in vitro* studies found that taxanes greatly increased the TP level in tumors, the combination of taxanes and capecitabine made synergic effect [26]. Therefore, we assumed that docetaxel might be a good companion for capecitabine in advanced UC.

Docetaxel is commonly used in platinum-resistant advanced UC, for which single-arm phase II trials reported RR of 13.3% and median OS of 9 months [6]. Previous clinical trials indicated docetaxel combined with oxaliplatin [27], gemcitabine [28, 29] or ifosfamide and cisplatin regimen [30] were mostly tolerable and moderately active (RR 27–47%) for advanced UC after failure of platinum-based therapy. Although in our study the ORR of DC (18.2%) was lower, the CBR and survivals were not inferior to prior results [3]. Patients with stable disease (SD) also benefitted from the treatment. We tried to evaluate the association of some factors with OS, like albumin. But we failed even in univariate analysis due to the limited sample size and immature OS data.

Our study showed that the AEs of capecitabine w/o docetaxel included anemia, leukopenia, mucositis, thrombocytopenia and HFS. Most were tolerable [6, 13]. Anemia and leukopenia were reported more in the DC arm than in the C arm, but the frequencies were not higher than former studies [13]. Severe leukopenia happened more in the DC arm than the C arm. But it was also manageable. No treatment-related death was observed in this study.

Recently Raggi et al. performed a meta-analysis to study the impact of single-agent compared with doublet chemotherapy as second-line therapy of advanced UC [31]. It showed that doublet regimen improved ORR and PFS significantly, while OS was not prolonged. And the toxicity was similar. When analyzing the regimens including taxanes (paclitaxel or docetaxel), only ORR advantage existed in the doublet therapy. Neither improvement of PFS nor OS was found. The authors recommend the clinical usage of single taxanes in the second-line setting. Another meta-analysis had different results. Sonpavde et al. found patients of combination chemotherapy showed improved OS compared with patients of single-agent chemotherapy as salvage therapy for advanced UC. The main limitation was that single and combination cohorts were derived from separate trials and not directly compared in prospective trials. Patients could receive combination chemotherapy might have better performance status [32]. According to our results, we had the same opinions on the usage of single-agent palliative chemotherapy. In some selected patients combination therapy might be an option.

There is a growing interest in targeted therapies in UC. However the results were not satisfied. For instance, even if epidermal growth factor receptor (EGFR) was overexpressed in the majority of UC, the response rates and survivals were disappointing in unselected population receiving gemcitabine and platinum regimen plus gefitinib [33, 34]. The application of bevacizumab in metastatic cases was proved marginally effective while inducing cardiovascular toxicity and treatment-related death [35, 36]. Overexpression or amplification in human epidermal growth factor receptor 2 (HER2) is variable in different population (20–50% of tumors) [37–39]. Two phase II studies containing trastuzumab and chemotherapy in metastatic patients revealed inconsistent results [40, 41]. Recently numerous genomic alterations are identified in UC, for example TP53, PI3K and FGFR3 [42, 43]. Related inhibitors are being studied as well. Immunotherapy is now the key focus of studies. CTLA-4 inhibitor, PD-1 inhibitors and PD-L1 inhibitors have shown compelling activities in advanced UC [44–46]. PD-L1 inhibitor atezolizumab (MPDL3280a) showed certain activity in patients with metastatic UC after failure of platinum –based chemotherapy. The RR for all patients was 15%. The activity was durable in responders. Patients with increased percentage of PD-L1-positive immune cells reached higher RR. Atezolizumab was safe and well tolerated in the patient population [47]. Due to the moderate efficacy of conventional chemotherapy, targeted therapy and immunotherapy may be the critical methods to improve the outcomes of patients with metastatic UC.

In conclusion, the retrospective study revealed that capecitabine w/o docetaxel was tolerable and might be an optional palliative treatment for metastatic UC. Although ORR was higher in the DC arm, the CBR, PFS and OS were not outstanding compared to the C single-drug regimen. Single-agent palliative treatment as docetaxel or capecitabine might be optional in pretreated patients with metastatic UC. Further randomized trials are warranted to validate the efficacy of capecitabine w/o docetaxel in metastatic UC.

## MATERIALS AND METHODS

### Study population

This study is a one-institution, observational, retrospective study that includes patients diagnosed with UC treated with capecitabine w/o docetaxel at Sun Yat-Sen University Cancer Center. Patients were treated with capecitabine w/o docetaxel as first-line or second-line therapy, between April 2009 and March 2015. Using our center-based database, all patients who received capecitabine between 2009 and 2015 and diagnosed of UC were identified (*n* = 49). Twelve patients were excluded because they received capecitabine for adjuvant chemotherapy. Seven patients without follow-up were excluded. One patient was excluded because he received capecitabine with oxaliplatin. Overall 29 patients were included in the analysis. If recurrence or metastasis occurred within 1 year after adjuvant chemotherapy, the regimen (capecitabine w/o docetaxel) was also defined as second-line therapy. Because no patient identification data was collected, and it was a retrospective descriptive study, moreover we did not have any interventions afterwards, so specific written or verbal informed consent was not provided to the participators.

### Treatments

All Patients were treated at a dose of capecitabine 1000mg/m^2^ given orally twice daily on days 1–14 every 3 weeks, among them 11 patients received docetaxel 75mg/m^2^ iv on day 1 every 3 weeks concurrently. The institutional ethics review board approved this study to review medical records including basic characteristics, prior therapy, capecitabine w/o docetaxel courses, efficacy, AEs, disease progression and death events.

Tumor response was evaluated by computed tomography (CT) or magnetic resonance imaging (MRI) every 2 cycles while in treatment period, according to RECIST 1.0. After progression, patients were followed up every 3 months until death. PFS was defined as the duration from the date of treatment began to the date of disease progression or last follow-up. OS was defined as the duration from the date of treatment began to the date of death from any cause or last follow-up. AEs were identified and recorded retrospectively. Severity of AEs was evaluated following Common Terminology Criteria for Adverse Events (CTCAE) version 4.0.

### Statistical analysis

The primary objective of this retrospective observational study was the CBR of capecitabine w/o docetaxel, including the percentage of complete response (CR), PR and SD. The secondary endpoints included AEs, PFS, and OS. PFS and OS were estimated using Kaplan–Meier methods and compared using the log-rank tests. Differences across treatment arms regarding all categorical variables were examined with a χ^2^ test. Analyses were carried out using the statistical software package SPSS 16.0(SPSS, Chicago, IL). All statistical tests were two-sided, and a *p*-value < 0.05 was considered as statistically significant.
